# *N*-(4,6-Dimethyl­pyrimidin-2-yl)-1*H*-benzimidazol-2-amine

**DOI:** 10.1107/S1600536811006507

**Published:** 2011-02-26

**Authors:** Shaaban Kamel Mohamed, Mahmoud A. A. El-Remaily, Atash V. Gurbanov, Ali N. Khalilov, Seik Weng Ng

**Affiliations:** a Chemistry & Environmental Science Division, School of Science, Manchester Metropolitan University, England; b Department of Chemistry, Sohag University, Sohag, Egypt; c Department of Organic Chemistry, Baku State University, Baku, Azerbaijan; d Department of Chemistry, University of Malaya, 50603 Kuala Lumpur, Malaysia

## Abstract

There are two independent mol­ecules in the asymmetric unit of the title compound, C_13_H_13_N_5_. In each mol­ecule, an amino N atom is connected to a benzimidazole fused-ring system and a pyrimidine ring [these are aligned at 1.3 (1)° in one independent mol­ecule and at 5.4 (1)° in the other]. The amino N atom of the fused ring forms an intra­molecular N—H⋯O hydrogen bond to a pyrimidine N atom in each mol­ecule. The amino N atom connecting the two ring systems inter­acts with the other N atom of the pyrimidine ring of an adjacent mol­ecule, generating centrosymmetric hydrogen-bonded dimers.

## Related literature

For the synthesis, see: Bossio *et al.* (1985 ▶); Shestakov *et al.* (2006 ▶).
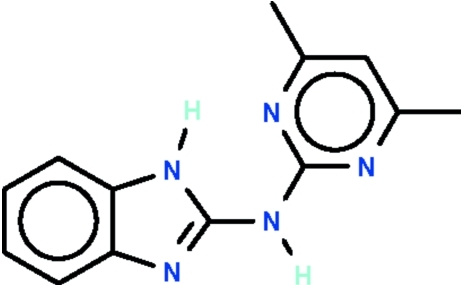


## Experimental

### 

#### Crystal data

C_13_H_13_N_5_
*M* *_r_* = 239.28
Triclinic, 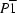

*a* = 8.2836 (4) Å
*b* = 9.6135 (5) Å
*c* = 16.5694 (8) Å
α = 92.121 (1)°
β = 96.100 (1)°
γ = 112.597 (1)°
*V* = 1206.94 (10) Å^3^
*Z* = 4
Mo *K*α radiation
μ = 0.09 mm^−1^
*T* = 295 K
0.30 × 0.20 × 0.20 mm

#### Data collection

Bruker APEXII diffractometer
13373 measured reflections
5550 independent reflections
4124 reflections with *I* > 2σ(*I*)
*R*_int_ = 0.019

#### Refinement

*R*[*F* ^2^ > 2σ(*F* ^2^)] = 0.044
*wR*(*F* ^2^) = 0.136
*S* = 1.01
5550 reflections
345 parameters
4 restraints
H atoms treated by a mixture of independent and constrained refinement
Δρ_max_ = 0.25 e Å^−3^
Δρ_min_ = −0.18 e Å^−3^

### 

Data collection: *APEX2* (Bruker, 2005 ▶); cell refinement: *SAINT* (Bruker, 2005 ▶); data reduction: *SAINT*; program(s) used to solve structure: *SHELXS97* (Sheldrick, 2008 ▶); program(s) used to refine structure: *SHELXL97* (Sheldrick, 2008 ▶); molecular graphics: *X-SEED* (Barbour, 2001 ▶); software used to prepare material for publication: *publCIF* (Westrip, 2010 ▶).

## Supplementary Material

Crystal structure: contains datablocks global, I. DOI: 10.1107/S1600536811006507/hg5003sup1.cif

Structure factors: contains datablocks I. DOI: 10.1107/S1600536811006507/hg5003Isup2.hkl

Additional supplementary materials:  crystallographic information; 3D view; checkCIF report

## Figures and Tables

**Table 1:** Hydrogen-bond geometry (Å, °)

*D*—H⋯*A*	*D*—H	H⋯*A*	*D*⋯*A*	*D*—H⋯*A*
N1—H1⋯N5	0.86 (1)	2.05 (2)	2.664 (2)	128 (2)
N3—H3⋯N2^i^	0.87 (1)	2.05 (1)	2.912 (2)	170 (2)
N7—H7⋯N10	0.87 (1)	2.10 (2)	2.695 (2)	125 (2)
N8—H8⋯N6^ii^	0.87 (1)	2.05 (1)	2.908 (2)	170 (2)

Symmetry codes: (i) 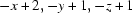; (ii) 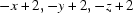.

**Fig. 1.**
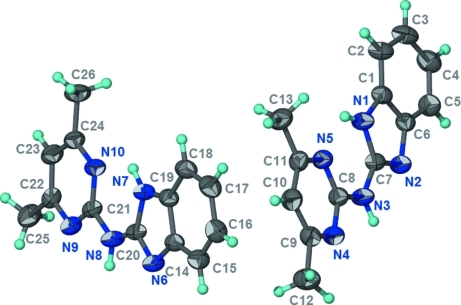
Thermal ellipsoid plot (Barbour, 2001) of the two independent molecules of C13H13N5 at the 50% probability level; hydrogen atoms are drawn as spheres of arbitrary radius.

## supplementary crystallographic information

### Comment

The reported synthesis involves the reaction of 4,6-dimethylpyrimidin-2-ylcyanamide with *o*-phenylenediamine, and it illustrates the type of heterocycles that are formed by the reaction of cyanamides with *N*,*N*-binucleophiles (Shestakov *et al.*, 2006). The unsubstituted compound was reported earlier (Bossio *et al.*, 1985). The present synthesis is a more convenient synthesis that uses acetylacetone as one of the reactants. An amino N atom in the approximately planar C_13_H_13_N_5_ molecule is connected to a benzimidazoyl fused-ring and a pyrimidyl ring; the amino N atom of the fused ring forms an intramolecular N–H···O hydrogen bond to a pyridmidyl N atom (Scheme I, Fig. 1). There are two independent molecules; each molecule is connected to an inversion-related molecule by an N–H···O hydrogen bond.

### Experimental

1*H*-Benzo[*d*]imidazol-2-yliminomethanediamine (0.05 mol) and acetylacetone (0.10 mol, approx.10 ml) along with several drops of acetic acid were heated at 473 for 1 h. The solid that formed on cooling was collected and recrystallized from ethanol to give the title compound in 80% yield; m.p. 623 K.

### Refinement

Carbon-bound H-atoms were placed in calculated positions [C–H 0.93 to 0.96 Å; *U*(H) 1.2 to 1.5*U*(C)] and were included in the refinement in the riding model approximation.

The amino H-atoms were located in a difference Fourier map, and were refined with a distance restraint of N–H 0.86±0.01 Å; their temperature factors were refined.

### Crystal data

**Table:**

C_13_H_13_N_5_	*Z* = 4
*M**_r_* = 239.28	*F*(000) = 504
Triclinic, *P*1	*D*_x_ = 1.317 Mg m^−^^3^
Hall symbol: -P 1	Mo *K*α radiation, λ = 0.71073 Å
*a* = 8.2836 (4) Å	Cell parameters from 4428 reflections
*b* = 9.6135 (5) Å	θ = 2.3–27.4°
*c* = 16.5694 (8) Å	µ = 0.09 mm^−^^1^
α = 92.121 (1)°	*T* = 295 K
β = 96.100 (1)°	Prism, colorless
γ = 112.597 (1)°	0.30 × 0.20 × 0.20 mm
*V* = 1206.94 (10) Å^3^	

### Data collection

**Table:**

Bruker APEXII diffractometer	4124 reflections with *I* > 2σ(*I*)
Radiation source: fine-focus sealed tube	*R*_int_ = 0.019
graphite	θ_max_ = 27.5°, θ_min_ = 1.2°
φ and ω scans	*h* = −10→10
13373 measured reflections	*k* = −12→12
5550 independent reflections	*l* = −21→21

### Refinement

**Table:**

Refinement on *F*^2^	Primary atom site location: structure-invariant direct methods
Least-squares matrix: full	Secondary atom site location: difference Fourier map
*R*[*F*^2^ > 2σ(*F*^2^)] = 0.044	Hydrogen site location: inferred from neighbouring sites
*wR*(*F*^2^) = 0.136	H atoms treated by a mixture of independent and constrained refinement
*S* = 1.01	*w* = 1/[σ^2^(*F*_o_^2^) + (0.0736*P*)^2^ + 0.1933*P*] where *P* = (*F*_o_^2^ + 2*F*_c_^2^)/3
5550 reflections	(Δ/σ)_max_ = 0.001
345 parameters	Δρ_max_ = 0.25 e Å^−^^3^
4 restraints	Δρ_min_ = −0.18 e Å^−^^3^

### Fractional atomic coordinates and isotropic or equivalent isotropic displacement parameters (Å^2^)

**Table:**

	*x*	*y*	*z*	*U*_iso_*/*U*_eq_	
N1	0.66435 (17)	0.61916 (15)	0.45010 (8)	0.0480 (3)	
N2	0.80016 (16)	0.45944 (14)	0.43969 (8)	0.0470 (3)	
N3	0.94151 (17)	0.67635 (15)	0.53228 (8)	0.0498 (3)	
N4	1.11489 (17)	0.88346 (14)	0.61816 (8)	0.0491 (3)	
N5	0.83527 (16)	0.86534 (14)	0.55242 (8)	0.0477 (3)	
N6	0.98905 (15)	1.11808 (14)	0.92502 (8)	0.0453 (3)	
N7	0.72463 (16)	1.09201 (14)	0.86230 (8)	0.0452 (3)	
N8	0.74067 (16)	0.92463 (15)	0.96388 (8)	0.0485 (3)	
N9	0.52809 (16)	0.73369 (14)	1.01630 (8)	0.0477 (3)	
N10	0.44770 (16)	0.87329 (14)	0.91397 (8)	0.0464 (3)	
C1	0.5574 (2)	0.50529 (17)	0.39234 (9)	0.0453 (3)	
C2	0.3985 (2)	0.48064 (19)	0.34608 (11)	0.0570 (4)	
H2	0.3415	0.5464	0.3516	0.068*	
C3	0.3283 (2)	0.3542 (2)	0.29138 (11)	0.0648 (5)	
H3A	0.2219	0.3343	0.2590	0.078*	
C4	0.4132 (2)	0.2562 (2)	0.28378 (11)	0.0646 (5)	
H4	0.3630	0.1726	0.2458	0.077*	
C5	0.5706 (2)	0.27942 (19)	0.33104 (10)	0.0566 (4)	
H5	0.6258	0.2122	0.3261	0.068*	
C6	0.64326 (19)	0.40638 (17)	0.38613 (9)	0.0446 (3)	
C7	0.80551 (19)	0.58625 (16)	0.47531 (9)	0.0433 (3)	
C8	0.96246 (19)	0.81387 (17)	0.56914 (9)	0.0444 (3)	
C9	1.1398 (2)	1.01836 (18)	0.65380 (10)	0.0502 (4)	
C10	1.0141 (2)	1.07950 (19)	0.64206 (10)	0.0541 (4)	
H10	1.0317	1.1723	0.6684	0.065*	
C11	0.8619 (2)	0.99939 (18)	0.59025 (10)	0.0492 (4)	
C12	1.3142 (2)	1.1021 (2)	0.70528 (13)	0.0679 (5)	
H12A	1.3969	1.0598	0.6921	0.102*	
H12B	1.2994	1.0930	0.7618	0.102*	
H12C	1.3577	1.2069	0.6949	0.102*	
C13	0.7188 (2)	1.0572 (2)	0.57189 (12)	0.0638 (5)	
H13A	0.6895	1.0528	0.5140	0.096*	
H13B	0.7591	1.1600	0.5946	0.096*	
H13C	0.6163	0.9959	0.5953	0.096*	
C14	1.01079 (19)	1.22562 (16)	0.86868 (9)	0.0423 (3)	
C15	1.1636 (2)	1.33597 (18)	0.84877 (10)	0.0510 (4)	
H15	1.2738	1.3468	0.8743	0.061*	
C16	1.1476 (2)	1.42941 (19)	0.78990 (10)	0.0563 (4)	
H16	1.2488	1.5041	0.7758	0.068*	
C17	0.9839 (2)	1.41438 (19)	0.75126 (10)	0.0569 (4)	
H17	0.9777	1.4789	0.7117	0.068*	
C18	0.8299 (2)	1.30521 (19)	0.77048 (10)	0.0525 (4)	
H18	0.7199	1.2954	0.7451	0.063*	
C19	0.84684 (19)	1.21133 (16)	0.82922 (9)	0.0433 (3)	
C20	0.81693 (18)	1.04241 (16)	0.91841 (9)	0.0417 (3)	
C21	0.56341 (18)	0.84116 (16)	0.96416 (9)	0.0433 (3)	
C22	0.3584 (2)	0.64850 (18)	1.01769 (10)	0.0489 (4)	
C23	0.2285 (2)	0.67368 (19)	0.96860 (11)	0.0554 (4)	
H23	0.1101	0.6147	0.9705	0.066*	
C24	0.27647 (19)	0.78683 (18)	0.91699 (10)	0.0494 (4)	
C25	0.3156 (2)	0.5228 (2)	1.07291 (12)	0.0647 (5)	
H25A	0.4053	0.5504	1.1191	0.097*	
H25B	0.3105	0.4326	1.0439	0.097*	
H25C	0.2036	0.5048	1.0911	0.097*	
C26	0.1440 (2)	0.8194 (2)	0.86077 (12)	0.0683 (5)	
H26A	0.1696	0.9257	0.8660	0.102*	
H26B	0.0280	0.7643	0.8746	0.102*	
H26C	0.1495	0.7889	0.8056	0.102*	
H1	0.653 (2)	0.6974 (15)	0.4721 (10)	0.062 (5)*	
H3	1.0279 (19)	0.647 (2)	0.5423 (11)	0.067 (5)*	
H7	0.6096 (12)	1.0539 (19)	0.8546 (11)	0.063 (5)*	
H8	0.811 (2)	0.902 (2)	0.9979 (9)	0.059 (5)*	

### Atomic displacement parameters (Å^2^)

**Table:**

	*U*^11^	*U*^22^	*U*^33^	*U*^12^	*U*^13^	*U*^23^
N1	0.0468 (7)	0.0444 (7)	0.0547 (8)	0.0227 (6)	−0.0035 (6)	0.0018 (6)
N2	0.0440 (7)	0.0451 (7)	0.0521 (7)	0.0199 (6)	−0.0010 (6)	−0.0007 (6)
N3	0.0447 (7)	0.0479 (7)	0.0585 (8)	0.0243 (6)	−0.0064 (6)	−0.0042 (6)
N4	0.0465 (7)	0.0477 (7)	0.0525 (7)	0.0203 (6)	−0.0013 (6)	−0.0007 (6)
N5	0.0470 (7)	0.0495 (7)	0.0504 (7)	0.0239 (6)	0.0032 (6)	0.0015 (6)
N6	0.0374 (6)	0.0460 (7)	0.0527 (7)	0.0178 (5)	−0.0004 (5)	0.0092 (5)
N7	0.0379 (6)	0.0476 (7)	0.0497 (7)	0.0186 (6)	−0.0035 (5)	0.0055 (5)
N8	0.0357 (6)	0.0484 (7)	0.0606 (8)	0.0167 (5)	−0.0021 (6)	0.0146 (6)
N9	0.0400 (6)	0.0491 (7)	0.0567 (8)	0.0203 (6)	0.0054 (6)	0.0072 (6)
N10	0.0383 (6)	0.0493 (7)	0.0498 (7)	0.0179 (5)	−0.0032 (5)	−0.0003 (6)
C1	0.0457 (8)	0.0438 (8)	0.0443 (8)	0.0157 (6)	0.0015 (6)	0.0101 (6)
C2	0.0526 (9)	0.0554 (9)	0.0616 (10)	0.0228 (8)	−0.0072 (8)	0.0119 (8)
C3	0.0567 (10)	0.0670 (11)	0.0595 (10)	0.0173 (9)	−0.0133 (8)	0.0074 (9)
C4	0.0641 (11)	0.0588 (10)	0.0566 (10)	0.0128 (9)	−0.0057 (8)	−0.0046 (8)
C5	0.0571 (10)	0.0504 (9)	0.0582 (10)	0.0182 (8)	0.0030 (8)	−0.0021 (7)
C6	0.0438 (8)	0.0451 (8)	0.0438 (8)	0.0160 (6)	0.0047 (6)	0.0079 (6)
C7	0.0415 (7)	0.0440 (8)	0.0456 (8)	0.0189 (6)	0.0020 (6)	0.0058 (6)
C8	0.0442 (8)	0.0441 (8)	0.0458 (8)	0.0187 (6)	0.0041 (6)	0.0032 (6)
C9	0.0507 (9)	0.0489 (8)	0.0486 (8)	0.0178 (7)	0.0029 (7)	0.0017 (7)
C10	0.0586 (10)	0.0495 (9)	0.0560 (9)	0.0245 (8)	0.0055 (8)	−0.0047 (7)
C11	0.0523 (9)	0.0510 (8)	0.0501 (8)	0.0259 (7)	0.0095 (7)	0.0035 (7)
C12	0.0601 (11)	0.0584 (10)	0.0767 (12)	0.0205 (9)	−0.0115 (9)	−0.0104 (9)
C13	0.0617 (11)	0.0672 (11)	0.0729 (12)	0.0382 (9)	0.0051 (9)	−0.0016 (9)
C14	0.0429 (7)	0.0425 (7)	0.0427 (7)	0.0196 (6)	0.0004 (6)	0.0015 (6)
C15	0.0464 (8)	0.0503 (9)	0.0527 (9)	0.0161 (7)	0.0018 (7)	0.0046 (7)
C16	0.0619 (10)	0.0485 (9)	0.0538 (9)	0.0158 (8)	0.0094 (8)	0.0069 (7)
C17	0.0740 (11)	0.0530 (9)	0.0490 (9)	0.0308 (9)	0.0052 (8)	0.0115 (7)
C18	0.0580 (9)	0.0567 (9)	0.0482 (9)	0.0306 (8)	−0.0026 (7)	0.0052 (7)
C19	0.0472 (8)	0.0428 (8)	0.0418 (7)	0.0218 (6)	−0.0007 (6)	−0.0004 (6)
C20	0.0381 (7)	0.0419 (7)	0.0466 (8)	0.0193 (6)	−0.0016 (6)	0.0028 (6)
C21	0.0370 (7)	0.0434 (8)	0.0503 (8)	0.0181 (6)	0.0009 (6)	−0.0005 (6)
C22	0.0431 (8)	0.0494 (8)	0.0560 (9)	0.0194 (7)	0.0101 (7)	0.0004 (7)
C23	0.0370 (8)	0.0599 (10)	0.0646 (10)	0.0145 (7)	0.0051 (7)	0.0024 (8)
C24	0.0372 (7)	0.0552 (9)	0.0527 (9)	0.0176 (7)	−0.0023 (6)	−0.0058 (7)
C25	0.0547 (10)	0.0660 (11)	0.0781 (12)	0.0242 (9)	0.0201 (9)	0.0210 (9)
C26	0.0422 (9)	0.0854 (13)	0.0718 (12)	0.0228 (9)	−0.0082 (8)	0.0089 (10)

### Geometric parameters (Å, °)

**Table:**

N1—C7	1.3535 (18)	C5—H5	0.9300
N1—C1	1.377 (2)	C9—C10	1.380 (2)
N1—H1	0.86 (1)	C9—C12	1.502 (2)
N2—C7	1.3176 (19)	C10—C11	1.376 (2)
N2—C6	1.3960 (19)	C10—H10	0.9300
N3—C7	1.3689 (19)	C11—C13	1.499 (2)
N3—C8	1.3756 (19)	C12—H12A	0.9600
N3—H3	0.87 (1)	C12—H12B	0.9600
N4—C9	1.336 (2)	C12—H12C	0.9600
N4—C8	1.3396 (19)	C13—H13A	0.9600
N5—C8	1.3343 (18)	C13—H13B	0.9600
N5—C11	1.340 (2)	C13—H13C	0.9600
N6—C20	1.3183 (18)	C14—C15	1.385 (2)
N6—C14	1.3917 (19)	C14—C19	1.397 (2)
N7—C20	1.3558 (18)	C15—C16	1.380 (2)
N7—C19	1.383 (2)	C15—H15	0.9300
N7—H7	0.87 (1)	C16—C17	1.388 (2)
N8—C20	1.3650 (19)	C16—H16	0.9300
N8—C21	1.3771 (19)	C17—C18	1.383 (2)
N8—H8	0.87 (1)	C17—H17	0.9300
N9—C22	1.3310 (19)	C18—C19	1.384 (2)
N9—C21	1.3367 (19)	C18—H18	0.9300
N10—C21	1.3352 (18)	C22—C23	1.380 (2)
N10—C24	1.3481 (19)	C22—C25	1.498 (2)
C1—C2	1.382 (2)	C23—C24	1.372 (2)
C1—C6	1.396 (2)	C23—H23	0.9300
C2—C3	1.378 (3)	C24—C26	1.496 (2)
C2—H2	0.9300	C25—H25A	0.9600
C3—C4	1.384 (3)	C25—H25B	0.9600
C3—H3A	0.9300	C25—H25C	0.9600
C4—C5	1.382 (2)	C26—H26A	0.9600
C4—H4	0.9300	C26—H26B	0.9600
C5—C6	1.386 (2)	C26—H26C	0.9600
			
C7—N1—C1	106.87 (13)	C9—C12—H12C	109.5
C7—N1—H1	120.2 (12)	H12A—C12—H12C	109.5
C1—N1—H1	132.8 (12)	H12B—C12—H12C	109.5
C7—N2—C6	104.12 (12)	C11—C13—H13A	109.5
C7—N3—C8	126.91 (13)	C11—C13—H13B	109.5
C7—N3—H3	115.8 (13)	H13A—C13—H13B	109.5
C8—N3—H3	117.0 (13)	C11—C13—H13C	109.5
C9—N4—C8	115.66 (13)	H13A—C13—H13C	109.5
C8—N5—C11	116.23 (13)	H13B—C13—H13C	109.5
C20—N6—C14	104.20 (12)	C15—C14—N6	129.94 (13)
C20—N7—C19	106.69 (12)	C15—C14—C19	119.88 (14)
C20—N7—H7	121.9 (12)	N6—C14—C19	110.18 (13)
C19—N7—H7	131.3 (12)	C16—C15—C14	118.09 (15)
C20—N8—C21	127.59 (13)	C16—C15—H15	121.0
C20—N8—H8	116.5 (13)	C14—C15—H15	121.0
C21—N8—H8	115.9 (12)	C15—C16—C17	121.51 (16)
C22—N9—C21	116.15 (13)	C15—C16—H16	119.2
C21—N10—C24	115.57 (14)	C17—C16—H16	119.2
N1—C1—C2	132.25 (15)	C18—C17—C16	121.25 (15)
N1—C1—C6	105.39 (13)	C18—C17—H17	119.4
C2—C1—C6	122.37 (15)	C16—C17—H17	119.4
C3—C2—C1	116.96 (16)	C17—C18—C19	116.95 (15)
C3—C2—H2	121.5	C17—C18—H18	121.5
C1—C2—H2	121.5	C19—C18—H18	121.5
C2—C3—C4	121.27 (16)	N7—C19—C18	132.51 (14)
C2—C3—H3A	119.4	N7—C19—C14	105.17 (12)
C4—C3—H3A	119.4	C18—C19—C14	122.32 (15)
C5—C4—C3	121.77 (16)	N6—C20—N7	113.75 (13)
C5—C4—H4	119.1	N6—C20—N8	122.52 (13)
C3—C4—H4	119.1	N7—C20—N8	123.73 (13)
C4—C5—C6	117.67 (16)	N10—C21—N9	127.34 (13)
C4—C5—H5	121.2	N10—C21—N8	118.60 (14)
C6—C5—H5	121.2	N9—C21—N8	114.06 (12)
C5—C6—C1	119.94 (14)	N9—C22—C23	120.91 (15)
C5—C6—N2	130.14 (14)	N9—C22—C25	117.17 (14)
C1—C6—N2	109.91 (13)	C23—C22—C25	121.90 (15)
N2—C7—N1	113.70 (13)	C24—C23—C22	119.11 (14)
N2—C7—N3	123.06 (13)	C24—C23—H23	120.4
N1—C7—N3	123.24 (14)	C22—C23—H23	120.4
N5—C8—N4	126.91 (14)	N10—C24—C23	120.91 (14)
N5—C8—N3	118.65 (13)	N10—C24—C26	116.69 (15)
N4—C8—N3	114.42 (13)	C23—C24—C26	122.39 (15)
N4—C9—C10	121.78 (15)	C22—C25—H25A	109.5
N4—C9—C12	116.46 (14)	C22—C25—H25B	109.5
C10—C9—C12	121.74 (15)	H25A—C25—H25B	109.5
C11—C10—C9	118.16 (15)	C22—C25—H25C	109.5
C11—C10—H10	120.9	H25A—C25—H25C	109.5
C9—C10—H10	120.9	H25B—C25—H25C	109.5
N5—C11—C10	121.22 (14)	C24—C26—H26A	109.5
N5—C11—C13	116.37 (15)	C24—C26—H26B	109.5
C10—C11—C13	122.40 (15)	H26A—C26—H26B	109.5
C9—C12—H12A	109.5	C24—C26—H26C	109.5
C9—C12—H12B	109.5	H26A—C26—H26C	109.5
H12A—C12—H12B	109.5	H26B—C26—H26C	109.5
			
C7—N1—C1—C2	−179.49 (17)	C20—N6—C14—C15	−179.64 (15)
C7—N1—C1—C6	0.26 (16)	C20—N6—C14—C19	0.28 (16)
N1—C1—C2—C3	178.38 (17)	N6—C14—C15—C16	−179.89 (15)
C6—C1—C2—C3	−1.3 (2)	C19—C14—C15—C16	0.2 (2)
C1—C2—C3—C4	0.4 (3)	C14—C15—C16—C17	0.0 (2)
C2—C3—C4—C5	0.7 (3)	C15—C16—C17—C18	0.2 (3)
C3—C4—C5—C6	−0.9 (3)	C16—C17—C18—C19	−0.6 (2)
C4—C5—C6—C1	0.0 (2)	C20—N7—C19—C18	−179.50 (16)
C4—C5—C6—N2	−178.51 (16)	C20—N7—C19—C14	0.38 (16)
N1—C1—C6—C5	−178.65 (14)	C17—C18—C19—N7	−179.37 (15)
C2—C1—C6—C5	1.1 (2)	C17—C18—C19—C14	0.8 (2)
N1—C1—C6—N2	0.17 (17)	C15—C14—C19—N7	179.52 (13)
C2—C1—C6—N2	179.95 (14)	N6—C14—C19—N7	−0.42 (16)
C7—N2—C6—C5	178.13 (16)	C15—C14—C19—C18	−0.6 (2)
C7—N2—C6—C1	−0.53 (16)	N6—C14—C19—C18	179.48 (14)
C6—N2—C7—N1	0.72 (17)	C14—N6—C20—N7	−0.03 (17)
C6—N2—C7—N3	−179.09 (14)	C14—N6—C20—N8	179.56 (13)
C1—N1—C7—N2	−0.64 (18)	C19—N7—C20—N6	−0.23 (17)
C1—N1—C7—N3	179.17 (14)	C19—N7—C20—N8	−179.82 (14)
C8—N3—C7—N2	176.24 (14)	C21—N8—C20—N6	177.63 (14)
C8—N3—C7—N1	−3.6 (3)	C21—N8—C20—N7	−2.8 (3)
C11—N5—C8—N4	−1.6 (2)	C24—N10—C21—N9	0.1 (2)
C11—N5—C8—N3	179.66 (14)	C24—N10—C21—N8	179.65 (13)
C9—N4—C8—N5	0.2 (2)	C22—N9—C21—N10	0.6 (2)
C9—N4—C8—N3	179.02 (13)	C22—N9—C21—N8	−178.95 (13)
C7—N3—C8—N5	3.8 (2)	C20—N8—C21—N10	1.4 (2)
C7—N3—C8—N4	−175.12 (15)	C20—N8—C21—N9	−179.03 (14)
C8—N4—C9—C10	1.5 (2)	C21—N9—C22—C23	−1.1 (2)
C8—N4—C9—C12	−176.69 (15)	C21—N9—C22—C25	177.35 (14)
N4—C9—C10—C11	−1.8 (3)	N9—C22—C23—C24	1.0 (2)
C12—C9—C10—C11	176.30 (16)	C25—C22—C23—C24	−177.42 (16)
C8—N5—C11—C10	1.2 (2)	C21—N10—C24—C23	−0.3 (2)
C8—N5—C11—C13	−179.73 (14)	C21—N10—C24—C26	−179.39 (14)
C9—C10—C11—N5	0.4 (3)	C22—C23—C24—N10	−0.2 (2)
C9—C10—C11—C13	−178.64 (16)	C22—C23—C24—C26	178.81 (16)

### Hydrogen-bond geometry (Å, °)

**Table:**

*D*—H···*A*	*D*—H	H···*A*	*D*···*A*	*D*—H···*A*
N1—H1···N5	0.86 (1)	2.05 (2)	2.664 (2)	128 (2)
N3—H3···N2^i^	0.87 (1)	2.05 (1)	2.912 (2)	170 (2)
N7—H7···N10	0.87 (1)	2.10 (2)	2.695 (2)	125 (2)
N8—H8···N6^ii^	0.87 (1)	2.05 (1)	2.908 (2)	170 (2)

Symmetry codes: (i) −*x*+2, −*y*+1, −*z*+1; (ii) −*x*+2, −*y*+2, −*z*+2.
